# Human papillomavirus and vaccination: knowledge, attitudes, and behavioural intention in adolescents and young women in Italy

**DOI:** 10.1038/sj.bjc.6604454

**Published:** 2008-07-15

**Authors:** G Di Giuseppe, R Abbate, G Liguori, L Albano, I F Angelillo

**Affiliations:** 1 Department of Public, Clinical and Preventive Medicine, Second University of Naples, Naples, Italy; 2 Chair of Hygiene, University of Naples ‘Parthenope’, Naples, Italy

**Keywords:** attitudes, behavioural intention, cervical cancer, human papillomavirus, Italy

## Abstract

This study assesses knowledge, attitudes, and behavioural intention towards human papillomavirus (HPV) infection and vaccination in a random sample of 1348 adolescents and young women aged 14–24 years in Italy. A self-administered anonymous questionnaire covered demographics; knowledge about HPV infection, cervical cancer, and HPV vaccine; the perceived risk for contracting HPV infection and/or for developing cervical cancer, the perceived benefits of a vaccination to prevent cervical cancer, and willingness to receive an HPV vaccine. Only 23.3% have heard that HPV is an infection of the genital mucosa and about cervical cancer. Those older, with at least one parent who is a health care professional, with personal, familiar, or friendly history of cervical cancer, and having underwent a health checkup in the last year with information about HPV vaccination were significantly more knowledgeable. Risk perception scores (range: 1–10) of contracting HPV infection and of developing cervical cancer were 5.8 and 6.5. Older age, not having a parent who is a health care professional, having had a personal, familiar, or friendly history of cervical cancer, and need of additional information were predictors of the perceived susceptibility of developing cervical cancer. The vast majority professed intent to receive an HPV vaccine and the significant predictors were having at least one parent who is a health care professional, a high perceived risk of contracting HPV infection and of developing cervical cancer, and a high belief towards the utility of a vaccination for preventing cervical cancer. Knowledge about HPV infection and cervical cancer should be improved with more attention to the benefit of HPV vaccination.

Human papillomavirus (HPV) is one of the most common sexually transmitted infections in sexually active adolescent girls and young women of several economically developed countries (Richardson *et al*, 2003; Syrjänen *et al*, 2005; Kitchener *et al*, 2006; Dunne *et al*, 2007). Strong evidence has been observed for the role of persistent high-risk HPV types (16 and 18), in the aetiology of cervical cancer, as worldwide they are responsible for approximately 70% of all cases (Clifford *et al*, 2006; Markowitz *et al*, 2007). Cervical cancer is the second most common cancer among women worldwide, with half a million who develop it annually and more than half of the cases die as a result (Parkin *et al*, 2005; Cutts *et al*, 2007).

Highly immunogenic and safe HPV vaccines recently have been licensed for use, the first explicitly designed to prevent cancer induced by a virus, which can reduce the morbidity and mortality of cervical cancer by offering protection to HPV types 16 and 18. Because the vaccine has the maximum benefit when given before a person becomes sexually active, the Advisory Committee on Immunization Practices of the Centers for Disease Control and Prevention (CDC) and the American Academy of Pediatrics, have recommended routine vaccination for girls aged 11 or 12 years (Clifford *et al*, 2006; American Academy of Pediatrics, 2007). In Italy, the Ministry of Health has recently approved a national programme for free HPV vaccination for girls aged 12 (Ministero della Salute, 2007).

Widespread acceptance of HPV vaccine is likely to provide enormous public health benefits (Garnett *et al*, 2006; Bosch *et al*, 2008; Goldhaber-Fiebert *et al*, 2008) and it must be administered before initiating sexual activity (Stanley *et al*, 2006; Markowitz *et al*, 2007; Saslow *et al*, 2007) and hence would be most effective if offered during early adolescence (Adams *et al*, 2007; Cutts *et al*, 2007). Because most sexually active individuals will become exposed to the virus throughout their lives and because the HPV vaccine is available, it is extremely important to gather data on HPV infection and vaccination. Such questioning has already taken place in several studies which mainly address vaccine acceptability among parents (Olshen *et al*, 2005; Brabin *et al*, 2006, 2007; Dempsey *et al*, 2006; Dinh *et al*, 2007; Marlow *et al*, 2007) and health care workers (Kahn *et al*, 2003, 2005; Aldrich *et al*, 2005; Riedesel *et al*, 2005; Moreira *et al*, 2006a, 2006b; Duval *et al*, 2007; Hopenhayn *et al*, 2007; Waller *et al*, 2007; Woodhall *et al*, 2007), but very little research has targeted adolescents. Therefore, this study attempts to generate information about knowledge, attitudes, and behavioural intention towards HPV infection and vaccination among adolescents and young women in Italy.

## Materials and methods

This cross-sectional investigation was conducted from March through May 2007 and the target population comprised 1348 women aged 14–24 years. The sample was selected with a two-stage cluster method. Seven faculties that cover a wide range of disciplines (economics, education, law, literature, and sport sciences) in two Universities and six public secondary schools in the geographic area of the Campania region, in the South of Italy, were randomly chosen. A simple random technique was adopted in selecting students from each faculty and school.

Before the study, a meeting with the Head and the Dean of each randomly selected school and faculty was arranged to present the project and the permission and collaboration were obtained. A questionnaire was pilot tested on 25 students before initiation of the survey, the objective of which was to identify the basic questions most valid for testing the comprehensiveness and relevance of our terms. As part of the process of informed consent, all potential candidates were invited to participate through a letter containing information about purpose and objectives, also indicating that participation in the survey was voluntary, and that privacy and confidentiality would be strictly protected as no personal identifiers were included in the questionnaire and data would be presented only in an aggregated manner. For those under 18 years of age, a separate information letter was addressed to the parents. These policies were also printed explicitly on the front page of the questionnaire. Provided that respondent and parental consent were obtained, data were collected by self-administered anonymous structured questionnaire administered with a reply envelope. The questionnaires were delivered to students in their classroom one time and were completed at the time of distribution. A respondent's consent was taken into account while filling the questionnaire. The questionnaire was used to explore several topics, including: (1) student characteristics, included age, marital status, employment status, whether they were living alone or not, and whether they have had a personal, familiar, or friendly history of cervical cancer; (2) knowledge about HPV infection (definition and modes of transmission), cervical cancer (definition and preventive measures), and availability of an HPV vaccine; (3) attitudes pertaining to assess whether or not the respondent perceived a risk for contracting HPV infection and/or for developing cervical cancer, opinions about the perceived benefits of a vaccination to prevent cervical cancer, and their willingness to receive an HPV vaccine; (4) behaviour, included questions whether or not respondents have ever had sex, age at first sex, number of acts of intercourse and of partners in the last year, and how frequently they were protected by condoms during intercourse with the partner(s) in the last year; health-promoting behaviour was assessed with questions about how often the respondent underwent a health checkup in the last year, whether respondents' health care provider informed them about HPV vaccine, and whether they have received the HPV vaccine; (5) sources of and needs of information regarding HPV vaccine and cervical cancer.

For items regarding their knowledge, participants were asked to provide answers with options for ‘no’, ‘do not know’, and ‘yes’. All attitudes were measured on a 10-point Likert scale with a score ranging from 1 to 10. The responses for the two questions about perceived risk were recorded as 1 for no risk at all and 10 for very high perceived risk; for the perceived benefits of a vaccination to prevent cervical cancer, 1 for not at all and 10 for high utility; and for their willingness to receive HPV vaccine, 1 for unwillingness and 10 for willingness. The respondents were also asked to report directly reason(s) for willingness or unwillingness to receive HPV vaccine. Questions pertaining to behaviours were close ended with nominal or categorical (yes or no) responses; the frequency of the use of condoms in the last year was measured on a five-point Likert scale ranging from ‘never’ to ‘every time’. Ethics committee approval was obtained.

### Statistical analysis

We analysed multivariable logistic and linear regression models using a forward stepwise procedure to assess the independent predictors of the following outcomes of interest: have heard about HPV infection and cervical cancer (Model 1); perception of risk of developing cervical cancer (Model 2); willingness to receive an HPV vaccine (Model 3). The following explanatory variables were included in all models: age (continuous), at least one parent is a health care professional (0=no, 1=yes), personal, familiar, or friendly history of cervical cancer (0=no, 1=yes), at least a health checkup in the last year and the physician informed them about HPV vaccination (0=no, 1=yes), number of sexual partners in the last year (0=0, 1=1, 2=⩾2), and need of information about HPV vaccination and cervical cancer (0=no, 1=yes). The following variables were also included: have already heard about HPV infection and cervical cancer (0=no, 1=yes) in Models 2 and 3; perception of risk of contracting HPV infection (continuous), perception of risk of developing cervical cancer (continuous), and perceived benefits of a vaccination to prevent cervical cancer (continuous) in Model 3. The significance level for variables entering the logistic and linear regression models was set at 0.2 and for removing at 0.4. Odds ratios (ORs) with 95% confidence intervals (CIs) were used in the logistic regression analyses. All reported *P*-values were assessed using two-sided tests and statistical significance was taken as a cutoff of *P*⩽0.05 throughout. The statistical package Stata (Version 8.1) was used for the analysis (Stata Corporation, 2003).

## Results

Subjects who participated in the survey were 1341 and a response rate of 99.5% was achieved. The mean age of participants was 19 years, almost all were single, the majority was full-time students, 8.9% had at least one parent who is a health care professional, and 16.3% has had a personal, familiar, or friendly history of cervical cancer.

The distribution of respondents who answered correctly to each of the different items of the knowledge about HPV infection and cervical cancer are presented in Table 1. Less than one-third (29.8%) reported that they have heard that HPV is one of the most common infections of the genital mucosa and three-quarters of them identified that the infection is primarily transmitted through sexual intercourse. Only half reported having heard of cervical cancer before and 60.2 and 34.8% of them, respectively, know that the majority of cases and deaths can be prevented through detection of precancerous changes in the cervix by using the Pap test and that condom use reduce the risk for HPV and cervical cancer. Less than half (42.1%) knows that the vaccine was a preventive measure, but only 15.3% knows that a vaccine is available in Italy. Overall, only 23.3% have heard that HPV is one of the most common infections of the genital mucosa and about cervical cancer. The adjusted ORs for the likelihood of having heard about HPV infection and cervical cancer by several variables are presented in Table 2. After multivariate logistic adjustments, this model reveals that older age was significantly associated with the outcome as the adjusted OR was 1.14 (95% CI 1.08–1.2) for every 1-year increment. This model also reveals that female subjects having at least one parent who is a health care professional (OR=1.66; 95% CI 1.07–2.58), having had a personal, familiar, or friendly history of cervical cancer (OR=3.04; 95% CI 2.26–5.11), and having underwent a health checkup in the last year and the physician informed them about HPV vaccination (OR=2.44; 95% CI 1.23–4.85) were associated with having higher odds of having heard about HPV vaccination compared to those who have not (Model 1 in Table 2).

Risk perception of contracting HPV infection and of developing cervical cancer was similar with the respondents inferring a level of perception with scores of 5.8 and 6.5, respectively and only 12.7 and 19.9% perceived themselves at a high risk, respectively by responding ‘10’. Table 2 presents the adjusted mean of perceived risk from a multiple linear regression model that controlled for the contribution of several variables. Older age, not having at least one parent who is a health care professional, having had a personal, familiar, or friendly history of cervical cancer, and need of additional information about this cancer and HPV vaccination were significantly the predictors of the perceived susceptibility of developing cervical cancer (Model 2 in Table 2). Subjects were asked about their belief towards the utility of an HPV vaccination for preventing cervical cancer and whether they would consider it in future, with a mean total score of 8.7 and 7.7, respectively. The vast majority (81.7%) professed intent to obtain an HPV vaccine in future. Multiple logistic regression was used to examine the probability of willingness to receive the HPV vaccination and the adjusted ORs are presented in Table 2. The significant predictors of the willingness to receive this vaccine were high perceived risk of contracting HPV infection (OR=1.18; 95% CI 1.08–1.29) and of developing cervical cancer (OR=1.09; 95% CI 1.01–1.18), a high belief in the utility of a vaccination for preventing cervical cancer (OR=1.33; 95% CI 1.24–1.42), and having one parent who is a health care professional (OR=1.86; 95% CI 1.03–3.39; Model 3 in Table 2).

For those who stated that they would consider the HPV vaccination in future, the main reasons were the belief that the vaccination reduces the risk of contracting HPV infection (73.8%) and of developing cervical cancer (59.7%), and felling at risk (10%). By contrast, for those who stated that they would not consider HPV vaccine, the most common reasons were the belief that the vaccine was dangerous (59.6%) and that they feel to be not at risk (42%).

According to sexual behaviour pattern, 45.4% reported current or previous sexual activity, and the mean age of first sexual intercourse of the sexually experienced was 17 years. Overall, only 27.1% of those sexually experienced reported that they have always used a condom during the intercourse in the last year. Approximately, half had at least one health checkup over the last year (48%) and for only 3%, the physician informed them about HPV vaccination.

When asked about their main source of information of HPV infection and cervical cancer, only 6% claimed health professionals. Among those who knew that a vaccine is available in Italy, 62.7 and 17.4% had received such information from television/mass media and from health professionals, respectively. Almost all respondents wanted more information about HPV vaccination (94.4%) and cervical cancer (95.7%).

## Discussion

This large study expands the previous reports and findings on knowledge, attitudes, and behaviour towards HPV infection, vaccination, and cervical cancer patterns among young women are of particular interest, because the responses reflect real issues in this population setting. This study included only women, mainly because when it has been conducted, the available HPV vaccine was approved for use in female subjects only and, as already indicated, international guidelines recommend vaccination of girls aged 11–12 years and in our country, it was free of charge only for girls of 12 years of age. Moreover, this approach is supported by cost–effectiveness analyses that have indicated that the additional vaccination of boys has a limited effect compared to girls-only vaccination (Taira *et al*, 2004; Newall *et al*, 2007).

Our results indicate that women's knowledge about HPV infection and cervical cancer was remarkably poor, as only 23.3% ever heard of them; particularly striking was that a very high proportion did not know that vaccination can prevent this cancer and that they were unaware of such a vaccine in Italy. This finding was lower than those observed in earlier surveys. Indeed, 33% of 397 adolescents, mean age of 15 years in Finland, have heard of HPV before addressing the questionnaire (Woodhall *et al*, 2007) and 58% of 811 university students in the United Kingdom have heard of HPV (Waller *et al*, 2007). Moreover, our prevalence that 75.2% knew about sexual transmission of HPV is higher than the 66.7% found in a study of 204 women aged 16–23 years attending a public outpatient gynaecological clinic in Brazil (Moreira *et al*, 2006a). As in these prior studies, our results suggest that the continued efforts are urgently needed to educate young women. As predicted, the concept of physician influence is clearly important in this context of educating and counselling patients and potentially it is at this level of initial contact between physician and patient when interventions designed to increase knowledge about HPV and cervical cancer might be best implemented. Having a physician visit for a checkup and information about HPV vaccination was an important predictor of a higher level of knowledge and it is also not surprising that we observed an association, although not reaching statistical significance, between the willingness to receive the vaccination and information from a physician. Indeed, there is a group of respondents who consider HPV vaccination in future simply if their physician engaged them in a discussion about HPV vaccine. However, if patients remain uneducated about the opportunities, then logically compliance and adherence with recommended vaccination will stay forever low. So it is reasonable that an endorsement from a trusted physician would most likely result in acceptance of HPV vaccination now that guidelines are in place and the vaccination is more popular.

Our study highlights the positive attitude of the sample, as a vast majority (81.7%) professed intent to receive the HPV vaccination. This finding is consistent with the already mentioned study in Finland where 86% declaring they would accept the vaccination (Woodhall *et al*, 2007); with a study in the United States, with 85% of participants 18–30 years of age, recruited from the community and clinical sites, who reported that they would be extremely or very likely to receive an HPV vaccine (Kahn *et al*, 2003). A lower value was reported in Brazil with 72% who would enroll in an HPV vaccine trial (Moreira *et al*, 2006a). Finally, in a population-based study in two Appalachian Kentucky counties, 92.1% of women aged 18–29 years indicated an interest in HPV vaccination (Hopenhayn *et al*, 2007).

Study participants showed that 12.7 and 19.9%, respectively, perceived themselves likely to acquire the infection and the disease by responding ‘10’. In the already mentioned studies of Brazil and United States, 68.1% perceived themselves at moderate-to-high risk (Moreira *et al*, 2006b) and 54% strongly or somewhat agreed that they were at risk of developing cervical cancer (Kahn *et al*, 2003). Further, the results of the logistic regression model showed that women who perceived a high risk of contracting HPV infection were more likely to receive the vaccination in future. That finding of an association between willingness to receive the vaccination and self-perceived risk of contracting infection agrees with a prior study (Moreira *et al*, 2006a). Why persons at higher self-perceived risk are the most willing to obtain vaccination is clear. Perceptions of heightened risk do serve as an effective stimulus to pursue vaccination. Another notable finding was that among the respondents who stated that they would not consider HPV vaccination in future, their most commonly stated reason appeared to be the belief that the vaccine was dangerous. These findings have implications for practice in public health as physicians, mainly those in primary care, are obligated to evaluate and rectify, when appropriate, any misperceptions or confusions about vaccination.

This study has some potential limitations. First, the research questions were investigated by a cross-sectional study design. Such design precludes determination of causal relationships between different factors and outcomes. Second, information was gathered by self-reported questionnaire and so the possibility exists that some responses may have reported incorrect information. Notwithstanding this criticism, the anonymity of the study may have reduced the variation in the responses. This study, however, displays important strengths: first, it provides information from a large number of participants and this allows exploration of very weak associations between variables; second, the data were essentially complete; third, the participation rate was very high, probably reflecting heightened interest for this survey.

In conclusion, our results suggest that the continuing efforts should be directed at focusing attention towards the benefits of following HPV vaccination recommendations emphasising those factors positively associated. There is also an urgent need for designing educational interventions to improve the level of knowledge about HPV infection and cervical cancer.

## Figures and Tables

**Table 1:** Knowledge about HPV and cervical cancer of the study population

	***N***	**%**
Have heard about HPV infection	399	29.8
Have heard about cervical cancer	706	52.6
		
	**Correct response**	
	***N***	**%**
*Modes of transmission of HPV* a		
Complete sexual intercourse (true)	300	75.2
Incomplete sexual intercourse (true)	116	29.1
Needle sharing (false)	91	22.9
Pregnancy (false)	70	17.5
Vaginal delivery (true)	63	15.8
		
*Preventive measures for cervical cancer* b		
Pap test (true)	425	60.2
Late start of sexual activity (true)	299	42.4
HPV vaccination (true)	297	42.1
Oral contraceptive (false)	256	36.3
Condom use (true)	246	34.8

HPV=human papillomavirus.

a Only for those who reported that they have heard about HPV infection.

b Only for those who reported that they have heard about cervical cancer.

**Table 2:** Logistic and linear regression models' results

**Variable**	**OR**	**SE**	**95% CI**	***P***
*Model 1. Have heard about HPV infection and cervical cancer. Log-likelihood*=−*648.78, χ*^*2*^=*104.86 (5 df), P*<*0.0001*				
Age	1.14	0.03	1.08–1.2	<0.001
Personal, familiar, or friendly history of cervical cancer	3.4	0.71	2.26–5.11	<0.001
Health checkup in the last year and the physician informed about HPV vaccination	2.44	0.86	1.23–4.85	0.011
At least one parent who is a health care professional	1.66	0.37	1.07–2.58	0.024
Number of sexual partners in the last year	1.24	0.14	0.99–1.55	0.062
				
*Model 3. Willingness to receive an HPV vaccine. Log-likelihood*=−*525.25, χ*^*2*^=*172.93 (6 df), P*<*0.0001*				
Perception of risk of contracting HPV infection	1.18	0.05	1.08–1.29	<0.001
Perceived benefits of a vaccination to prevent cervical cancer	1.33	0.05	1.24–1.42	<0.001
Perception of risk of developing cervical cancer	1.09	0.04	1.01–1.18	0.03
At least one parent who is a health care professional	1.86	0.57	1.03–3.39	0.041
Health checkup in the last year and the physician informed about HPV vaccination	2.92	1.7	0.93–9.16	0.065
Number of sexual partners in the last year	0.88	0.11	0.69–1.12	0.291
				
**Variable**	**Coefficient**	**SE**	***t***	***P***
*Model 2. Perception of risk of developing cervical cancer. F (5,1281)*=*8.98, P*<*0.0001, R*^*2*^=*3.4*% *adjusted R*^*2*^=*3*%				
Need of information about cervical cancer and HPV vaccination	1.66	0.36	4.54	<0.001
Age	0.06	0.02	2.61	0.009
Personal, familiar, or friendly history of cervical cancer	0.59	0.27	2.16	0.031
At least one parent who is a health care professional	−0.53	0.26	−2.02	0.043
Have heard about cervical cancer	0.2	0.16	1.25	0.212
Constant	3.55			

CI=confidence interval; HPV=human papillomavirus; OR=Odds ratio; SE=standard error.
